# Coral architecture affects the habitat choice and form of associated gobiid fishes

**DOI:** 10.1007/s00227-013-2354-x

**Published:** 2013-11-19

**Authors:** Lucien Untersteggaber, Philipp Mitteroecker, Juergen Herler

**Affiliations:** 1 Department of Integrative Zoology, Faculty of Life Sciences, University of Vienna, Althanstrasse 14, 1090 Vienna, Austria; 2 Department of Theoretical Biology, Faculty of Life Sciences, University of Vienna, Althanstrasse 14, 1090 Vienna, Austria

## Abstract

Gobiid fishes of the genus *Gobiodon* live in strong association with certain reef-building corals that vary considerably in size and architecture. These fishes hence are excellent model systems for studying evolutionary adaption to specific microhabitats. Using a sample of *Gobiodon histrio* and *G. rivulatus* and their most important host corals (*Acropora digitifera* and *A. gemmifera)* from the northern Red Sea, we assess (1) how corals that are occupied by gobies differ in their architecture from colonies that are not occupied and (2) how fish body shape is associated with the architecture of their host coral. Fish body shape was assessed by geometric morphometric techniques. Coral measurements included colony size, branch length (BL), and interbranch as well as branch tip distance of adjacent branches, for which we applied a new and non-destructive measurement technique based on casts of two-component epoxy resin. The most important factor influencing the occupation of corals was a BL of more than 5 cm. The distance between coral branches was clearly related to the width of the fishes and hence constrained overall fish size. *G. histrio* and *G.* *rivulatus* differ in adult body shape as well in their allometric development of lateral body compression, resulting in different maximum body sizes attainable in the restricted interbranch space of corals. The strong dependence of coral-associated fishes on large coral colonies with specific architectures increases the extinction risk of fishes within deteriorating coral reefs.

## Introduction

Adaptation to a specific habitat plays a central role in the evolution and diversification of organisms. Within coral reefs, the great diversity of coral forms and growth types (Wallace 1999; Veron 2000) offers various ecological niches for occupying organisms. An important microhabitat in branching corals is the space between the branches. Several groups of closely related animal species occupy these narrow ecological niches, including invertebrates (e.g., crabs) and fishes (e.g., gobies and damselfishes). They have evolved a great morphological diversity and hence are excellent models for studying adaptation and habitat specialization.

The complex architecture of corals provides retreat areas for fishes and other organisms against predators, nursery grounds, as well as food resources (Lassig 1981; Beukers and Jones 1998; Almany 2004a; Almany 2004b). This makes corals important constituents of biodiversity (Doherty and Sale 1986; Roberts and Ormond 1987; Hixon and Beets 1993; Roberts et al. 2002; Hobbs and Munday 2004; Munday 2004). The architecture of coral colonies, in turn, depends on their exposure to wave movement and other environmental factors (Bradbury and Young 1981). Their structure is therefore critically affected by environmental change.

Among coral-associated fishes, gobies represent the most species-rich group (Munday and Jones 1998). Gobies are very abundant in many ecosystems and occupy a great range of habitats, in particular among coral reefs (Miller 1996; Munday and Jones 1998; Herler 2007). Most gobies have a small body size and have evolved an extraordinarily high diversity (Nelson 2006). The decrease in body size often provides access to new, spatially constrained environments and thus to new trophic levels in the food chain, even in a saturated ecosystem (Miller 1979; Munday and Jones 1998). The body shape of coral-occupying gobies (e.g., genus *Gobiodon*) is assumed to be adapted to their most preferred host corals. In particular, narrow interbranch distances (IBDs) may favor more compressed and deeper body shapes (Herler et al. 2007), but empirical evidence for these assumptions is scarce. For example, in experiments at the Great Barrier Reef, *Gobiodon histrio*, which has a highly specialized body shape, grew slower and suffered higher mortality in a suboptimal coral compared to its preferred host coral, whereas survival of the less specialized *G. brochus* was similar in both corals (Munday 2001; Munday et al. 2001).

In the northern Red Sea, most of the nine species of *Gobiodon* occupy only a few species of *Acropora* and show little overlap in habitat use (Dirnwöber and Herler 2007; Herler 2007; Herler et al. 2013). The two species *G. histrio* and *G. rivulatus* are exceptions, both of which preferably occupy the host coral *Acropora digitifera*, and, at a lower frequency, also *A. gemmifera*. *G. histrio* grows larger, yielding a superior rank in the competitive hierarchy among species, and therefore occupies the preferred coral *A. digitifera* more frequently. This species also shows high habitat specialization and coral host fidelity, both pointing to adaptations for minimizing post-settlement migration (Munday et al. 2001; Dirnwöber and Herler 2007; Wall and Herler 2009).

In this paper, we study variation in body shape in species of the coral-associated genus *Gobiodon* in relation to host coral architecture. We focus on the two species *G. histrio* and *G. rivulatus* and on their two main host corals *A. digitifera* and *A. gemmifera*. The similar habitat choice of the two *Gobiodon* species leads to interspecific competition (Dirnwöber and Herler 2007): A larger body size would lead to a higher rank in the competitive hierarchy (Munday 2001) but also to decreased adaptation to the narrow interbranch space of the corals. We thus addressed the following questions: (1) How do occupied coral colonies differ in their architecture from colonies that are not selected by gobies? (2) How is fish body shape related to host coral architecture? (3) Do the spatial constraints for fishes inhabiting the corals’ interbranch space result in shape differences between fish species with a different body size?

## Materials and methods

### Coral morphology

**Fig. 1:**
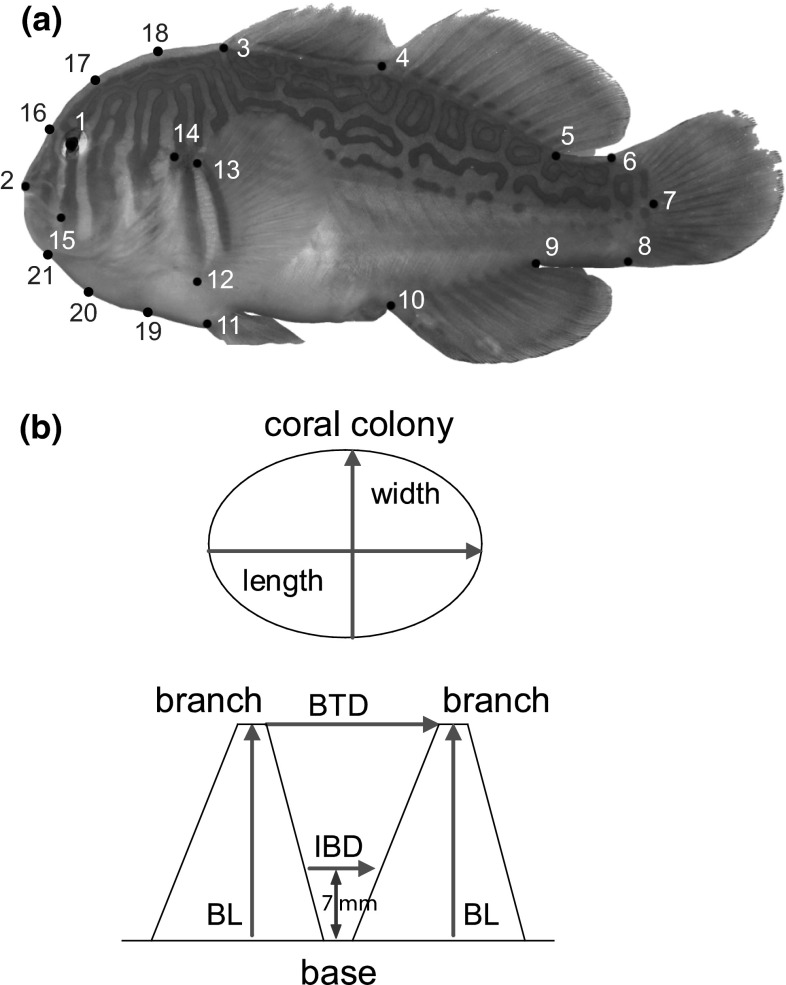
**a** Landmarks used for geometric morphometric analysis of *Gobiodon* from the northern Red Sea. Landmark definitions are given in Table 1. Landmarks *16*–*21* are sliding semi-landmarks, placed equidistantly between two landmarks. **b** Coral architecture measurements: *BTD* branch tip distance (from the center of the axial corallite of one branch to that of the adjacent branch), *BL* branch length of two adjacent branches from the branch tip (axial corallite) to the base (deepest point between two main branches), *IBD* interbranch distance (7 mm above the base of two adjacent branches)

We sampled data from corals and fishes in a shallow water reef (“Napoleon Reef”) in the Gulf of Aqaba, northern Red Sea at Dahab, Egypt (28°28′N, 34°30′E), in April 2010. A total of 53 colonies of *A. digitifera* (*n* = 27) and *A. gemmifera* (*n* = 26), from the reef flat and the reef edge, were examined. Of these colonies, 19 were occupied and 34 were unoccupied. Maximum colony length (L) and width (W) were measured to the closest 1 cm using a reference ruler. These two measures were used to approximate the mean colony diameter (L + W)/2. As proxies for coral branch architecture, two dimensions of coral branches were measured (Fig. 1b). Branch length (BL) of two adjacent branches was measured from the branch tip (the axial corallite) to the base (the deepest point between two main branches) using the depth gauge of a plastic calliper. Branch tip distance (BTD) between the same two branches was measured as the distance from the center of the axial corallite of one branch to that of the other branch. Both dimensions were measured to the closest mm for ten randomly selected branch pairs per colony.

Most *Gobiodon* species live in the innermost space of corals (Herler 2007). To measure the IBD close to basis of the branches, a new and non-destructive measurement technique was applied to the two coral species. Custom-made forceps (anatomical forceps DP11, with a grooved jaw profile) of two different lengths (145 and 300 mm) were grinded over most of their length prior to their use in the field to facilitate insertion between even narrowly spaced coral branches. A two-component epoxy resin (Reef Construct™, Aqua Medic GmbH) was mixed at the study site immediately before diving. This epoxy was then used to create casts of coral branch bases: A cylindrical piece of still soft epoxy (approximately 2 cm long and 0.3 cm wide) was inserted into the coral using the forceps and pressed against the base of two adjacent branches. Every piece of epoxy, before usage, was size-adjusted by hand to ensure that it will fill the corals’ interbranch space to a minimum height of 1.5 cm. The casts were carefully removed and stored in small, subdivided, and numbered plastic boxes to preserve their shape. The casts hardened within 2 h and were then measured in the laboratory with a digital calliper. The grooved jaw profile on the cast was useful for re-establishing a similar holding position (approximately perpendicular to the measuring axis of the IBD) of the forceps. Tiny imprints of radial corallites of the two coral branches in the cast also helped in finding the correct measuring position. Cast width was measured at a height of 7 mm from the base with a digital calliper to the closest 0.01 mm. We chose a height of 7 mm because adult specimens of *Gobiodon* on average have a body depth (dorso-ventral height) of 14 mm and hence have their maximum body width at approximately 7 mm height.

### *Gobiodon* morphology

We assessed body shape in subadult or adult preserved specimens of *G. histrio* (*n* = 33, SL > 22 mm) and *G. rivulatus* (*n* = 21, SL > 21 mm) from the same reef in the northern Red Sea. All specimens were fixed in 5 % formalin and preserved in 70 % ethanol. The thick epidermal mucus layer, typical for *Gobiodon*, was scraped off with a scalpel to reveal the anatomical points (landmarks) required for morphometric analysis.

A geometric morphometric (GM) approach was used to study shape differences between species of *Gobiodon*. All specimens were scanned with an EPSON Perfection 4990 flatbed scanner using a 3,200 dpi resolution, according to the protocol of Herler et al. (2007). A set of 15 anatomical landmarks and 6 sliding semi-landmarks per specimen (Fig. 1a; Table 1) was digitized on the randomized images using the program tpsDig 2.14 and tpsUtil 1.44 (Bookstein 1997; Rohlf 2009a; Rohlf 2009b). The landmark configurations were standardized for location, orientation, and scale by General Procrustes Analysis (Rohlf and Slice 1990). The resulting Procrustes shape coordinates were used for statistical analysis and visualization of shape differences.

**Table 1:** Description of 15 landmarks and 6 semi-landmarks (SLM; equidistantly placed between two landmarks) for geometric morphometric analyses

Landmark	Description
1	Center of orbit
2	Anterior tip of snout
3	Anterior insertion of first dorsal fin
4	Anterior insertion of second dorsal fin
5	Posterior insertion of second dorsal fin
6	Dorsal insertion of caudal fin
7	Midpoint of origin of caudal fin
8	Ventral insertion of caudal fin
9	Posterior insertion of anal fin
10	Anterior insertion of anal fin
11	Insertion of ventral fin
12	Ventral insertion of pelvic fin
13	Dorsal insertion of pelvic fin
14	Dorsal origin of operculum
15	Most posterior point of lips
16–18	SLM on forehead (between LM 2 + 3)
19–21	SLM along chest (between LM 2 + 11)

In addition to the GM approach, we further measured body depth (Vd; dorso-ventral body height at ventral fin origin) and greatest head width (gHw; widest horizontal transversal head dimension) in a subsample of similar-sized (<28 mm SL) *G. histrio* (*n* = 16) and of all *G. rivulatus* (*n* = 21). The body volume was approximated from these two variables together with standard length (SL) by calculating the volume of an ellipsoid SL × Vd × gHw × *π* × 0.166; the lateral body display area was approximated as SL × Vd × *π* × 0.25.

We assessed the relationship between coral morphology and fish shape using another sample of live fish (21 adult *G. histrio*, 9 adult *G. rivulatus*) taken from the occupied corals. Fishes were taken from colonies of *A. digitifera* and *A. gemmifera* with clove oil (Munday and Wilson 1997), allowed to recover, and stored in numbered plastic boxes. In the laboratory, fishes were kept in a 160-l aquarium and supplied with fresh seawater by a flow-through system. Fishes were narcotized again with clove oil, following the protocol of Munday and Wilson (1997), and laterally scanned on an EPSON Perfection V30 flatbed scanner using a 3,200 dpi resolution, following the protocol of Herler et al. (2007). SL and body depth were measured on the scans, whereas greatest head width (gHw; widest head dimension), head width (Hw; distance between the left and right upper opercular insertions), and greatest body width (gBw; widest body dimension) were directly measured on narcotized fishes with a digital calliper to the closest 0.01 mm. After scanning and taking measurements, fishes were allowed to recovered in aerated seawater and released back to the reef.

### Statistical analysis

We compared the variables mean colony diameter (mean DIA), BL, BTD, and IBD at 7 mm height across occupied and unoccupied colonies of *A. digitifera* and *A. gemmifera* by three-way ANOVAs with occupation status, coral species, and reef zone as independent factors. Multivariate differences in these groups were explored by a principal component analysis (PCA) of the log-transformed measurements. To identify aspects of coral morphology that are most important for coral occupation by gobies, we performed a logistic regression of occupation status (0, 1) on the log-transformed variables BL, BTD, IBD, and mean DIA. These analyses were computed with Mathematica 8.0 and SPSS 17.0.

In order to explore shape differences between the *Gobiodon* species, PCA was applied to the Procrustes shape coordinates of the 33 *G. histrio* and 21 *G. rivulatus* specimens using the software tpsRelw 1.49 (Rohlf 2010) and PAST 2.03 (Hammer et al. 2001). A MANOVA was performed on the first seven principal component scores with PAST 2.03 to test for group mean differences.

An ANCOVA was used to test for species differences in allometric relationships between the variables body depth, greatest head width, body volume, and lateral body display area.

To identify relationships between the shape of fishes and that of host corals, a two-block partial least squares (2B-PLS) analysis (Sampson et al. 1989; Streissguth et al. 1993; Rohlf and Corti 2000) was performed on the log-transformed coral measurements (BL, BTD, IBD, DIA) and fish measurements (gBw, gHw, Hw, SL, Vd; 21 *G. histrio* and 9 *G. rivulatus*) with the program PAST 2.03. Two-block PLS yields linear combinations (latent variables) for each block with maximum covariance between the two sets of variables (in this case, fish measurements and coral measurements).

## Results

### Morphology of occupied and unoccupied coral colonies

**Fig. 2:**
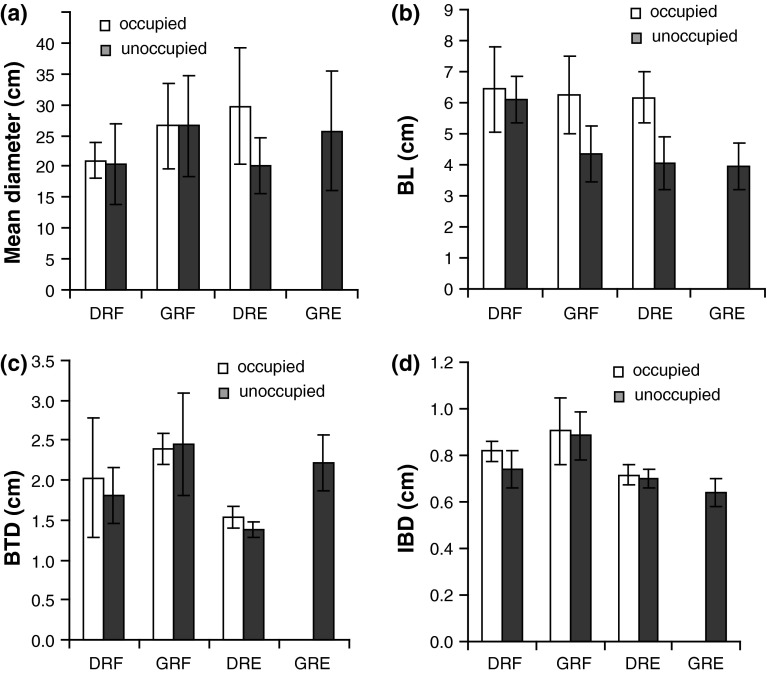
Coral morphometrics. Two occupied (*n* = 24) and unoccupied (*n* = 35) coral species [*A. digitifera* (D) and *A. gemmifera* (G)] were measured at two different reef zones—reef flat (RF) and reef edge (RE). Measurements include **a** mean colony diameter, **b** branch length (BL), **c** branch tip distance (BTD), and **d** interbranch distance (IBD) at 7 mm height. Values are means ± standard deviations

Mean colony diameter (Fig. 2a) of all occupied colonies was larger (mean ± SD = 25.5 ± 7.5 cm, *n* = 19) than that of unoccupied colonies (23.7 ± 8.1 cm, *n* = 34). Mean BL was larger (Fig. 2b) in occupied (6.3 ± 1.1 cm) than in unoccupied colonies (4.7 ± 1.2 cm), whereas mean BTD of occupied colonies (1.98 ± 0.57 cm) was slightly smaller than in unoccupied (2.07 ± 0.55 cm) colonies (Fig. 2c). Mean IBD at 7 mm height (Fig. 2d) of occupied colonies (0.81 ± 0.11 cm) was larger than in unoccupied colonies (0.75 ± 0.12 cm). Three-way ANOVA showed that BL and to some degree also IBD differ significantly between occupied and unoccupied colonies even when accounting for species differences and differences between the reef zones (Table 2).

**Table 2:** Results of three-way ANOVAs with occupation status, species, and reef zone as independent factors and branch length (BL), branch tip distance (BTD), interbranch distance (IBD), and mean colony diameter (mean DIA) as dependent variables

	Occupation status	Species	Reef zone
BL	*p* = 0.0001	*p* < 0.0001	*p* = 0.0007
BTD	*p* = 0.6602	*p* < 0.0001	*p* = 0.0182
IBD	*p* = 0.0129	*p* = 0.0587	*p* < 0.0001
Mean DIA	*p* = 0.1603	*p* = 0.0857	*p* = 0.2791

All pairwise interaction terms were not significant, except for reef zone × species with IBD as dependent variable (*p* = 0.0014)

**Fig. 3:**
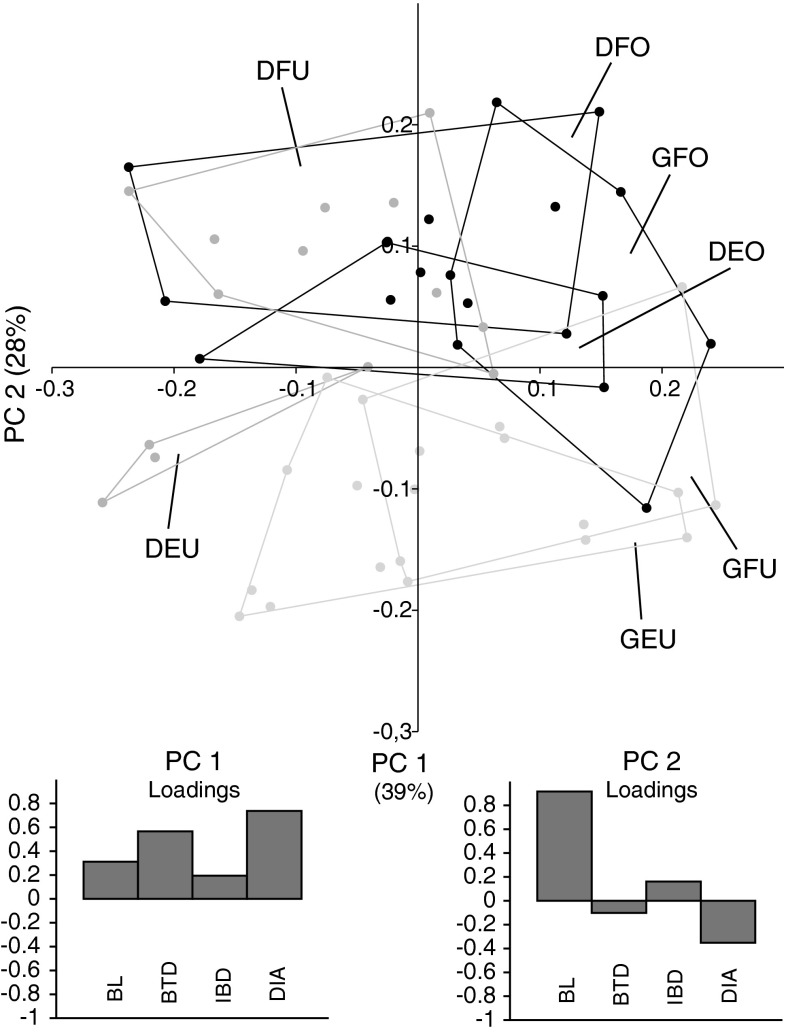
Scatter plot and loadings of PC 1 and PC 2 of the log-transformed variables *BL*, *BTD*, *IBD* at 7 mm height, and mean colony diameter of occupied (O) and unoccupied (U) *A.* *digitifera* (D) and *A. gemmifera* (G) colonies from the reef flat (F) and reef edge (E). Loadings of variables are indicated for *PC 1* and *PC 2*

The PCA of BL, BTD, IBD, and mean colony diameter (Fig. 3) showed that colonies of *A. digitifera* were separated from *A. gemmifera* mainly along PC 1, whereas PC 2 separated occupied colonies from unoccupied colonies (with the exception of unoccupied *A. digitifera* from the reef flat). The high loading of BL on PC 2 indicates an important role of BL for the coral’s occupation status.

Logistic regression of occupation status on BL, BTD, IBD, and mean colony diameter likewise revealed that BL was the most important variable (regression coefficient = 16.3; *p* < 0.001) to promote occupation. The effect of IBD on occupation was only barely significant (regression coefficient = 17.4, *p* < 0.05).

### Body shape variation in *Gobiodon*

**Fig. 4:**
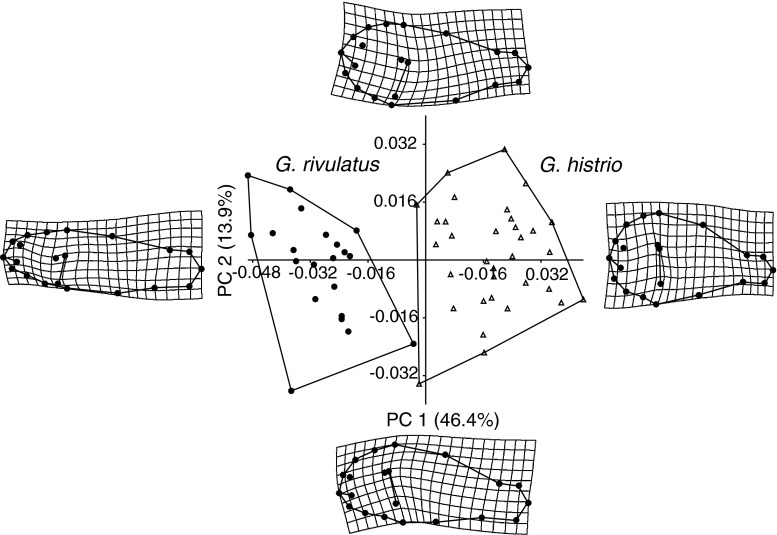
*Scatter plot* and deformation grids of the first two principal components (% of explained variances in parentheses) of Procrustes shape coordinates of *Gobiodon* *histrio* (*n* = 33) and *G. rivulatus* (*n* = 21). The deformation grids visualize shape deformations along principal components (deformations correspond to 0.1 unit deviation from the mean shape along each axis)

**Fig. 5:**
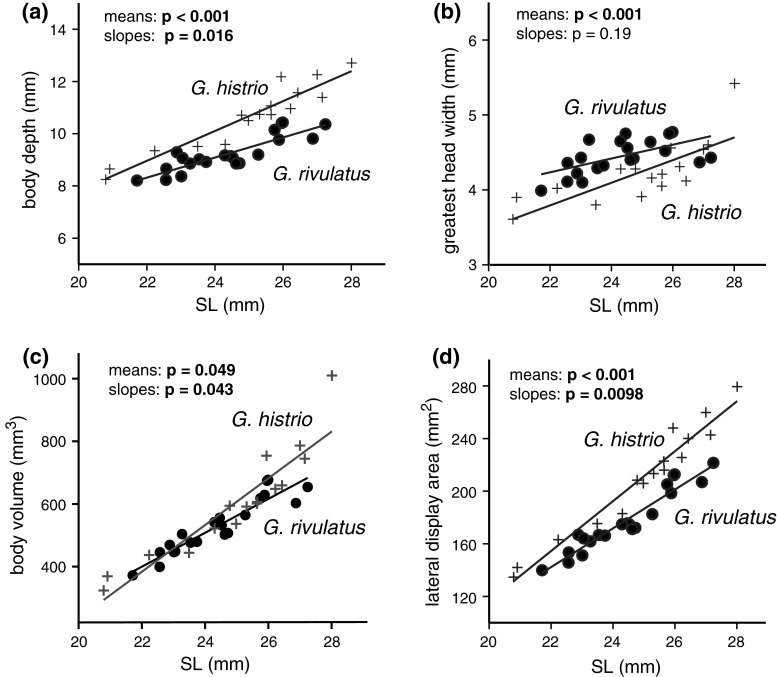
*Scatter plot* of **a** body depth at ventral fin origin, **b** greatest head width, **c** body volume, and **d** lateral body display area against standard length (SL) in similar-sized preserved *G. histrio* (*n* = 16) and *G. rivulatus* (*n* = 21) specimens. The *p*- values of the one-way ANCOVA for differences in adjusted means and regression slopes are indicated; significant ones are in *bold*

The PCA of Procrustes coordinates revealed a clear phenotypic separation between *G. histrio* and *G. rivulatus*, with no overlap along PC 1 (Fig. 4). *G. rivulatus* differs from *G. histrio* by a lower body depth, longer head, lower position of the pectoral fin base, and a larger caudal peduncle area. Average SL did not differ significantly between the subsample (<28 mm SL) of *G.* *histrio* (24.9 ± 2.1 mm, *t* test: *p* = 0.37, *n* = 16) and all *G*. *rivulatus* (24.4 ± 1.5 mm, *n* = 21). Body depth was higher in *G. histrio* (10.6 ± 1.3 mm) than in *G. rivulatus* (9.2 ± 0.7 mm), and ANCOVA showed significantly different adjusted means (*p* < 0.001). By contrast, greatest head width was significantly smaller in *G. histrio* (4.2 ± 0.4 mm) than in *G. rivulatus* (4.5 ± 0.2 mm; *p* < 0.001). The increase in Vd with SL (regression slope) was higher in *G. histrio* than in *G. rivulatus* (*p* = 0.016), whereas the increase in gHw was not significantly different between the two species (*p* = 0.19) (Fig. 5). Interestingly, despite distinct differences in average body depth (*p* < 0.001) and average greatest head width, average body volume was similar in *G. histrio* and *G. rivulatus* (601 ± 172 vs 529 ± 88 mm^3^; ANCOVA: *p* = 0.049; *t* test: *p* = 0.1; Fig. 5c), indicating that the body volume remains relatively constant across different shapes. Lateral body display size was higher on average in *G. histrio* than in *G. rivulatus* (210 ± 41 vs 177 ± 23 mm^2^; *p* < 0.01) and increased faster with size (homogeneity of slopes: *p* < 0.01; Fig. 5d). Thus, *G*. *histrio* is deeper-bodied (on average 44 % vs 38 % of SL) but narrower-headed (16.9 vs 18.3 % of SL) and consequently has a more laterally flattened shape than *G. rivulatus*.

### Relation between coral architecture and fish body shape

**Fig. 6:**
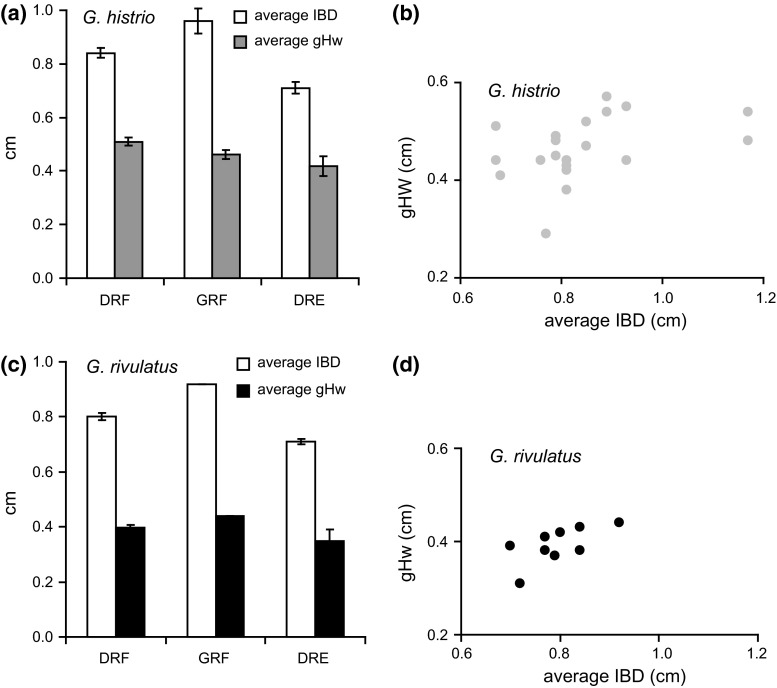
IBD measurements of *Acropora digitifera* (D) and *A. gemmifera* (G) occupied by *G. histrio* (*n* = 21) and *G. rivulatus* (*n* = 9) from two reef zones (reef flat (RF) and reef edge (RE)), and respective fish head width measurements shown in *bar charts* (**a**, **c**), and plotted against each other (**b**, **d**). Values in *bar charts* are means ± standard errors

Two-block partial least squares analysis of coral and fish measurements (both log-transformed) yielded one dimension (pair of latent variables) accounting for 96.7 % of squared covariance between the two sets of variables. The loadings of the coral measurements (BL 0.19, BTD 0.89, IBD 0.17, mean diam. 0.37) were dominated by BTD, whereas the loadings of the fish measurements were all relatively high (SL 0.46, gHw 0.34, Vd 0.63, Hw 0.36, gBw 0.38). This indicates that the relationship between coral architecture and fish morphology was driven by the association between BTD and overall fish size (particularly SL and Vd). A weak association also exists between IBD and gHw (Fig. 6). The average gHw of all *G. histrio* specimens was about half the average IBD of their host corals.

## Discussion

Coral morphology plays a major role in reef habitat complexity (Almany 2004a; Almany 2004b) and influences the community structure of associated organisms (Chabanet et al. 1996. Vytopil and Willis (2001) described that tightly branched coral species show a greater abundance and species richness of epifauna compared to open-branched species. This suggests that protection afforded by complex habitats is important in structuring the communities of coral-associated organisms. They also showed that interbranch space significantly affected the size of different species of *Tetralia* crabs when they were associated with different coral species. Wall and Herler (2009) revealed frequent habitat change in small single adult *G. histrio* individuals, indicating that they move around some time to find their ideal breeding coral. After finding a suitable coral, migration rates drop and a strong relationship is established, even leading to a defense against coral predators and competitors (Dirnwöber and Herler 2013; Dixson and Hay 2012). This shows that coral-associated invertebrates and fishes select host corals based on their architecture and that juveniles and subadults are forced to change colonies as they grow larger.

The present study revealed differences in architecture between occupied and unoccupied coral colonies. Occupied colonies at the reef edge were significantly larger than unoccupied ones, agreeing with a previous study in the same area (Schiemer et al. 2009). Large-sized colonies seem to be preferred because a larger shelter and breeding ground, combined with a greater food resource, are ideal conditions for the occupation of such spatially restricted microhabitats. BL was higher in occupied colonies and appeared to be the most important feature of coral architecture affecting goby occupation. The improved shelter effect of long-branched (>5 cm) colonies promotes inhabitation, whereas short-branched colonies are rarely inhabited because settled fishes are either quickly removed by predators, or already avoid such corals during habitat selection—a behavior for which strong selective pressure can be expected. As a consequence, the small-sized and especially the short-branched *A. gemmifera* colonies at the reef edge were never occupied (see Fig. 2).

Physical stress on coral architecture through wave movement affects colony shape and can be a relevant factor for coral occupation. Munday et al. (1997) suggested that fishes in general avoid zones with high hydrodynamic stress, but we found that preferably shaped *A. digitifera* colonies in the high-energy zone (reef edge) were frequently occupied by gobies. By contrast, several *A. digitifera* colonies on the less exposed reef flat with clearly preferable architecture were unoccupied. Previous studies report occupation rates of approximately 80 % among certain species of *Acropora* (Patton 1994; Munday and Jones 1998; Dirnwöber and Herler 2007), and there is an inverse relationship between coral abundance and occupation rates of *Gobiodon* spp. in each reef zone. Schiemer et al. (2009) found the highest coral and fish density on the reef flat, and the highest occupation rate for goby breeding pairs (about 41 %) occurred in *A. digitifera*. Moreover, in the present study, relatively large but unoccupied *A. digitifera* colonies had to be sampled selectively because the frequency of large unoccupied colonies was extremely low. Therefore, the most likely explanation for the emptiness of these theoretically suitable corals is that such colonies were actually waiting for fishes to move in, or that unknown factors prevented gobies from selecting them.

Apart from morphological differences between occupied and unoccupied corals, interspecific differences were found. The two coral parameters mean colony area and BTD clearly differed between the two coral species examined. BTD was lower in *A. digitifera*, which may, in combination with similar BLs, favor a higher occupation rate of this clearly most suitable host coral (Schiemer et al. 2009). The two-block partial least squares analysis of occupied colony architecture and fish morphology revealed a connection between BTD and fish size, suggesting that colonies with wider branching support larger fishes, which require more space. Larger fishes do also need less protection from relatively small predators, which would be able to access widely branched corals.

Fish body size was positively correlated with the lateral compression of the body, i.e., larger fishes were relatively thinner, which is the only way to evolve increased body size in the constrained coral interbranch space. IBD at 7 mm height above the coral branch base was associated with fish length and head width, indicating that a minimum interbranch space may be necessary for successful inhabitation. IBD can thus be interpreted as a “filter” for fish size (and shape) by restricting the greatest body width, or, as is the case in the genus *Gobiodon*, greatest head width. The more specialized habitat choice of *G. histrio* is also reflected in its more specialized shape. In particular, body depth has a strong positive allometric component in *G*. *histrio*, whereas head width was smaller than in *G. rivulatus*. This results in a higher maximum length and lateral body display area in *G*. *histrio*. Body length is important during turf wars, but the lateral appearance plays the major role (Collyer et al. 2005). Therefore, the growth pattern of *G. histrio* may be highly favorable in a guild where interspecific competition for habitats is high (Munday et al. 2001); a large body size or lateral display area will help gain a superior competitive rank (Collyer et al. 2005; Wong et al. 2007, 2008).

As coral inhabitants with a cylindrical body shape cannot grow large in the constrained interbranch space, the question arises why did not all species have evolved a larger but more compressed body? One possible explanation is that the use of a wider array of host corals with a different architecture prevents adaptation to a particular coral geometry. A less specialized body shape, as found, for example, in *G. rivulatus*, *G. reticulatus*, and *G*. sp.1, is typically associated with a more generalistic habitat choice (Herler 2007; Dirnwöber and Herler 2007). In our sample, differences in body shape did not affect body volume. The relatively constant volume across different shapes indicates that internal organs are more likely to change their shape than their volume. However, extreme body compression may negatively affect vital organs or functions, such as egg bestowal or locomotion.

Less specialized body shapes of small species may also result from high interspecific competition, in which small species are subordinate and forced to use alternative habitats, such as narrow-branched corals, which are unsuitable for the larger and dominant species. Such corals will in turn strongly limit the maximum body size of associated fishes and hence avoid competition. Examples include the very small gobies *G.* sp.2 (Herler and Hilgers, 2005) and *G. prolixus*, which inhabit very narrow-branched corals (Herler, personal observations; Winterbottom and Harold 2005). By contrast, species of *Gobiodon* that occupy corals other than *Acropora* seem to have experienced less selective pressure to develop extreme body shapes or small sizes. *Gobiodon winterbottomi*, for example, which lives in the plate-like coral *Echinopora lamellosa*, has a notably larger head width of about 19.3 % of SL (calculated from Suzuki et al. 2012) than the similar-sized *G. histrio* (16.9 % of SL).

Habitat specialization is also reflected in the behavior of coral-associated fishes. Although both *G. histrio* and *G. rivulatus* show considerable overlap in habitat use, *G. histrio* has a clear preference for *A. digitifera*, in which the highest breeding pair frequency is established (Schiemer et al. 2009). The smaller maximum body size of *G. rivulatus* leads to lower success in interspecific competition for habitats and thus to a more frequent use of alternative coral hosts (Dirnwöber and Herler 2007). As there is a trade-off between competition success and fitness in suboptimal corals, however, it is expected that the fitness consequences for living in such corals are less for *G. rivulatus* (Munday 2001). By contrast, the fitness of *G. histrio* suffers significantly when inhabiting less optimal corals, and therefore, this species competes strongly for its most preferred host coral (Munday 2001, Hobbs and Munday 2004). *G. histrio* even exhibits mutualistic behavior, which includes efficient defense of the coral against corallivorous fishes and algae (Dixson and Hay 2012; Dirnwöber and Herler 2013). It furthermore supports its host coral by showing a lower level of corallivory and higher ingestion of algal overgrowth (Riedlecker and Herler 2009; Brooker et al. 2010).

In summary, we show that coral architecture is strongly related to the successful occupation by coral-associated reef fishes and to the fishes’ body form. A more generalistic habitat choice behavior is reflected by a less specialized body shape. We further demonstrated a trade-off between body shape and body size within the same habitat. The findings are ecologically important because the strong dependence of coral-associated fishes on large coral colonies with specific architectures increases the extinction risk of fishes within deteriorating coral reefs. Increased frequencies of reef bleaching events particularly affect the most important host coral genus *Acropora* (McClanahan et al. 2008), which is likely to lead to a decreased species richness and average colony size. Smaller corals will have shorter branches and a narrower IBD and may be unsuitable for occupation by obligate associates. This will especially affect larger fish breeding pairs, which maintain the reproductive success and population size of their species.
